# Editorial: Into the uncharted territory of XR in second/foreign language education: psychological contributors and barriers

**DOI:** 10.3389/fpsyg.2023.1215548

**Published:** 2023-06-22

**Authors:** Lindsay Miller, Junjie Gavin Wu, Danyang Zhang, Nathan Thomas

**Affiliations:** ^1^Department of English, City University of Hong Kong, Kowloon, Hong Kong SAR, China; ^2^School of Foreign Languages, Shenzhen Technology University, Shenzhen, China; ^3^College of International Studies, Shenzhen University, Shenzhen, China; ^4^Centre for Applied Linguistics, University College London, London, United Kingdom

**Keywords:** extended reality, augmented reality, virtual reality, mixed reality, XR, language education, innovative learning, gaming

Nowadays, we expect technology to be an integral part of our lives, socially, professionally, and in education. However, it is worth noting that there were several significant milestones in getting to where we are today in education. For instance, the introduction of the radio in the 1920s into the classroom allowed teachers to bring the world alive by allowing their students to listen to real-time broadcasts. Then, in the early 1930s, overhead projectors allowed teachers to move away from writing on a blackboard and to prepare lessons in advance with the use of acetates. Video recorders and tapes appeared in the 1950s, which helped liven up classroom learning, enabling teachers to go beyond the textbooks and motivate students in their learning via technology. In the 1970s, we saw two significant technological innovations: the handheld calculator and the Scantron machine. The low cost of small calculators allowed students to perform mathematics in their seats in short time. The Scantron was a method whereby multiple-choice tests could be easily marked. This time-saving device allowed for more standardized testing and more reliability in the results of tests. As we entered the 1980s, and with the great leaps in technology, laptops and smartboards all became available to the teacher and students in the classroom. Of course, each of these technological devices were enhanced with the introduction of the internet in the early 1990s.

Each aspect of the introduction of technology into the classroom had a significant knock-on effect on pedagogy and the way students learned (see Hafner and Miller, 2019). It should also be noted that as each new technology became available for teachers to use, there were detractors objecting to the use of such technology in the classroom. As we allow students to make use of their smartphones to check for information or bring an iPad into the classroom, there are those who complain that such devices lead to cheating or make the students lazy to think things out for themselves. Interestingly, the same is the case today whenever a new technology is used for teaching and learning. However, as is also the case, with the introduction of each new technology into the classroom we have moved forward in our understanding of how to make use of the technology and not allow the technology to control us. It comes as no surprise then that as we continue to progress further into the 21^st^ century, the strides in technology for educational purposes have advanced.

The latest major technological innovation to enter into education is the use of extended reality (XR), which is rapidly transforming the way we teach and learn languages. XR is an umbrella term that includes any type of virtual reality (VR), augmented reality (AR), or mixed reality (MR) technologies which help students experience the contexts in which they need to make use of a second or foreign language (Wu et al., 2023). Similar to how radio, television and other technologies in the past tried to enliven language lessons, XR is another step in the attempt to make real-life contexts of classroom-based learning. One example of the use of XR is that used in the Metaverse, where students can choose an avatar to engage in business meetings, conferences and social gatherings to immerse themselves in the target language environments and practice their language skills. Such applications extend the learning experience beyond the classroom context and what traditional teaching methods could achieve.

In this volume, we introduce four papers which deal with innovations in technology that have an impact on teaching and learning in the classroom. The first paper by Zhi and Wu examines XR-based lessons. In this paper, the authors use the Cognitive Affective Model of Immersive Learning to examine previous studies to explain how such a model is of use in the classroom. The outcome of this analysis shows that the authentic and immersive environment can be extremely useful to the advancement of learners' cognitive dimensions, together with linguistic development.

In the second paper, Xiao and He examine students' language learning experiences via a 3D digital game. Test results between the experimental and control groups showed the effectiveness of digital gaming as the experimental group had better performance in all aspects of their language learning after game-based learning than the control group. However, the researchers caution that such improvements are not achieved overnight and that long-term planning (i.e., pedagogical innovation) of how to use gaming for language learning is needed, which includes an understanding of gaming principles, and how the elements of gaming can be exploited in an educational setting.

Paper three in this volume is by Feng and Ng. In their paper, the authors focus on how VR technology can help EFL learners overcome difficulties they may have when developing their writing in English. The study presented here focuses on improving vocabulary and writing performance by students who learned with immersive virtual reality (IVR) compared to those who learned via traditional classroom-based instruction. The results show that students who used IVR performed significantly better on word usage, lexical density, distribution richness and completion of tasks than their counterparts who were taught writing by more traditional teaching methods.

In the final article, Hua and Wang present a detailed review of virtual reality-assisted language learning from 2008–2022, and bring us up-to-date with the situation so far. These authors comment that the rapid development of VR and new technologies has increased the number of publications and the trends of how VR might be used for education. The results of these publications are that the scope of VR in education is expanding, and the benefits and drawbacks of using VR in the classroom are becoming better known. Based on their review, Hua and Wang present some implications for practitioners and researchers in their article.

The studies presented in this volume show the interest and exciting possibilities for using XR and other new technologies for language learning purposes. The potential for XR to transform pedagogical and language learning is significant, and although we are only at the frontier of such a radical change to education, it is a change we cannot ignore. As technology continues to develop, we can expect more changes to how teachers teach and learners learn. Innovative approaches to language education, by way of extended reality applications, is the future.

## Author contributions

LM drafted the editorial. JW, DZ, and NT reviewed and edited the editorial. All authors contributed to the article and approved the submitted version.
